# [^18^F]SiTATE PET for PRRT selection and monitoring metastatic tumors of the adrenal medulla and extra-adrenal paraganglia

**DOI:** 10.1007/s00259-025-07550-2

**Published:** 2025-09-19

**Authors:** Sophie C. Siegmund, Adrien Holzgreve, Magdalena Schöll, Vera U. Wenter, Gabriel T. Sheikh, Maximilian Scheifele, Franz Josef Gildehaus, Simon Lindner, Matthias K. Auer, Christian Lottspeich, Matthias Kroiß, Svenja Nölting, Friederike Völter, Christine Spitzweg, Christoph J. Auernhammer, Rudolf A. Werner, Mathias J. Zacherl

**Affiliations:** 1 Department of Nuclear Medicine, LMU University Hospital, LMU Munich, Marchioninistr. 15, 81377 Munich, Germany; 2 Bavarian Cancer Research Center (BZKF), partner site, Munich, Germany; 3 Ahmanson Translational Theranostics Division, David Geffen School of Medicine at UCLA, Los Angeles, CA USA; 4 Department of Internal Medicine IV, LMU University Hospital, LMU Munich, Munich, Germany; 5 Endocrinology and Diabetology, Schweinfurt, Germany; 6 Department of Endocrinology, Diabetology and Clinical Nutrition, University Hospital Zurich and University of Zurich, Zurich, Switzerland; 7 The Russell H Morgan Department of Radiology and Radiological Sciences, Division of Nuclear Medicine, Johns Hopkins School of Medicine, Baltimore, USA

**Keywords:** (3–6): [^18^F]SiTATE, Peptide receptor radionuclide therapy (PRRT), Paraganglioma, Pheochromocytoma, Somatostatin receptor (SSTR)

## Abstract

**Purpose:**

In somatostatin receptor (SSTR)-expressing tumors, theranostics with SSTR-directed imaging and therapy showed promising results regarding disease control. This study evaluated the use of PET imaging with [^18^F]SiTATE in pheochromocytoma and paraganglioma (PPGL) patients, focusing on eligibility for peptide radioreceptor therapy (PRRT) and therapy monitoring.

**Methods:**

Five patients with metastatic paraganglioma (n = 3) or pheochromocytoma (n = 2) were included. Eligibility for PRRT was assessed by [^18^F]SiTATE applying the Krenning score and baseline SUV_max_. Treatment response was analyzed by RECIST 1.1 criteria, total tumor volume (PET-based TTV), and Chromogranin A (CgA).

**Results:**

At baseline, all patients showed high lesional uptake, with the highest in the bone (mean SUV_max_ 41.4 ± 87.3) and a high Krenning Score of 3–4, Suggestive for PRRT eligibility. At the follow up, 2.5 months after completion of PRRT, all patients presented with stable disease (RECIST 1.1) and decreasing or stable CgA levels, whereas TTV increased in three patients and thus showed heterogenous response.

**Conclusion:**

In metastatic PPGL, [^18^F]SiTATE effectively visualizes tumor burden and supports patient selection and response assessment for PRRT. Notably, the data revealed a heterogenous response across PET-based, CT-based, and biochemical assessments. The underlying mechanisms of these discrepancies remain unclear and warrant further investigation.

## Introduction

Rare neuroendocrine neoplasms include tumors of the adrenal medulla (e.g. pheochromocytoma (PCC)) or tumors of the extra-adrenal paraganglia (e.g. paraganglioma (PGL)) [1, 2]. Both tumor subtypes, hereinafter PPGL to include both entities, can develop into malignant tumors in < 1 to 79% of cases, as the progression is very heterogeneous and depends on tumor size and genotype, among other factors [3, 4]. Tumor cells express Chromogranin A (CgA) [2], which is furthermore co-stored and secreted with catecholamines [5]. [^18^F]SiTATE is a novel radiotracer beyond established ^68^Ga-labeled SSTR-directed tracers, that can be not only used in the imaging of somatostatin receptors (SSTR) expressing tumor entities, but also in a theranostic setting to evaluate PRRT eligibility, as reported for other SSTR-expressing tumor entities [6–9]. Due to the widespread SSTR expression, PPGLs are suitable candidates for targeted radionuclide therapy when other therapeutic options are either not feasible or ineffective [10–14].

This study evaluated the use of SSTR-targeted imaging with the novel tracer [^18^F]SiTATE in PPGL patients, focusing on eligibility for PRRT and therapy monitoring through changes in tumor volume, RECIST 1.1 criteria and hormone levels.

## Materials and methods

### Study design and patients

This retrospective, observational study was conducted under the provisions of the German Medicines Products Act § 13(2b) accordance with the tenets of the Declaration of Helsinki and after approval by the Institutional Ethics Committee of the Ludwig-Maximilians-Universität Munich (IRB #21–0102 and #24–0982).

#### PRRT and imaging

[^18^F]SiTATE was prepared as described elsewhere and injected intravenously at a mean activity of 225 ± 33 MBq [6, 15, 16].

Patients underwent baseline PET to assess PRRT eligibility. 2.5 months after the second PRRT cycle, a follow-up PET was performed to assess treatment response. PRRT ([177Lu]Lu-DOTATATE) was administered intravenously at a mean activity of 7455 ± 191 MBq, following established dosing recommendations [17, 18].

Depending on the outcome, the further course of therapy was individualized: in cases of good response and high therapeutic need (e.g., high tumor burden), additional PRRT cycles were administered promptly. In the event of disease progression, treatment was discontinued. As part of a “rechallenge” strategy additional PRRT cycles were given later once disease progression occurred [19, 20]. In case of additional PRRT cycles, a further follow-up imaging assessment was performed approximately 2.5 months after completion of the PRRT treatment.

All PET/CT scans were performed at the Department of Nuclear Medicine, LMU Munich, using a Biograph mCT Flow scanner or a Biograph 64 PET/CT (Siemens Healthineers, Erlangen, Germany) as described previously [8]. Contrast-enhanced diagnostic CT scans were acquired in the portal venous phase using iopromide (Ultravist-300, Bayer Healthcare, Leverkusen, Germany) at a dose of 1.5 mL per kg body weight. Image reconstruction was performed as described previously [8].

PET was analyzed using dedicated software (Hermes Hybrid Viewer, Affinity 1.1.4; Hermes Medical Solutions, Stockholm, Sweden). The maximum standardized uptake values (SUV_max_) of the hottest 20 SSTR positive lesions per organ system (lymph node, bone, lung, liver) as well as potential primary or local recurrence were determined. Tracer uptake was visually analyzed using the Krenning score in order to determine PRRT eligibility [21].

Treatment response was assessed by the total tumor volume (TTV) on PET, using an SuV threshold of 4.0 [8]. Stable TTV was defined as ± 30%, progression as + 30% and response as −30%.

CT scans were evaluated using RECIST 1.1 criteria with dedicated software (mint lesion™, version 3.8.6, Mint Medical GmbH, Dossenheim, Germany) [22, 23].

Imaging parameters were compared with CgA levels.

Tumor control was defined as stable or decreasing TTV or CgA levels or stable disease (SD) according to RECIST 1.1.

### Statistical analysis

Data analysis was conducted descriptively using Microsoft Excel (Excel 2019, Microsoft, Redmond, WA, USA). Results are presented as mean ± standard deviation (StD).

## Results

### Patient characteristics

**Fig. 1:**
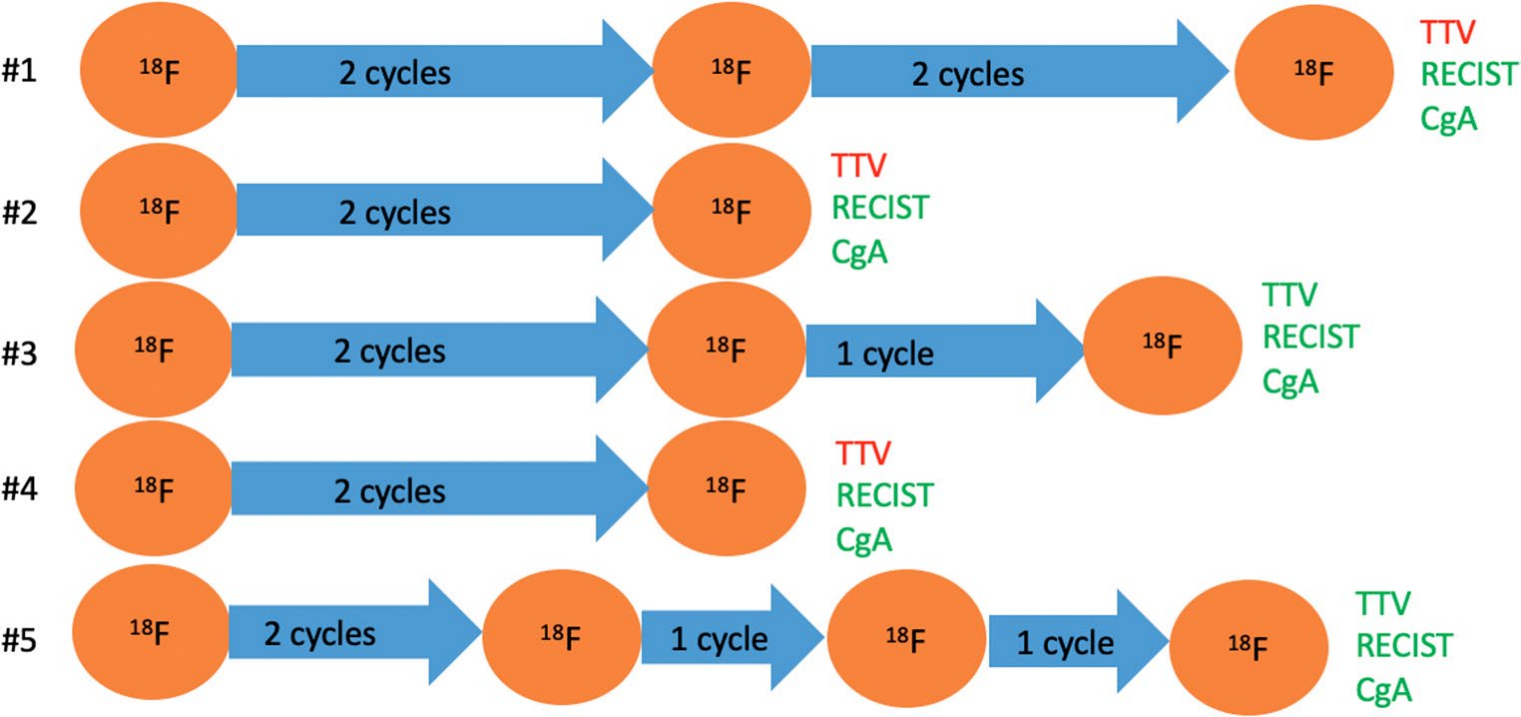
Overview of imaging and therapy regimens: All patients presented with Stable disease according to RECIST 1.1 criteria and decreasing or stable CgA levels. In contrast TTV increased in three patients and was stable or decreasing in two patients. CgA Chromogranin A; TTV total tumor volume; * green letters: tumor control; red letters: progression

Five patients were included, 3/5 (60.0%) with PGL and 2/5 (40.0%) with PCC (mean age 47 ± 17 years; 2 female/3 male). Systemic pretreatments are displayed in Table 1. Treatment regimens and imaging are displayed in Fig. 1.

**Table 1:** General patient characteristics

ID	Sex	Age	Subtype	Genetic cluster	Mutation	Ki67 [%]	Biochemical phenotype	Systemic pretreatment	Mx pattern
1	F	50	PCC	sporadic	no	10	noradrenergic		LN, O
2	M	55	PCC	sporadic	no	n.a.	adrenergic	9 cycles of cyclophosphamid, dacarbazin, vincristin	LN, O, PUL, P
3	M	30	PGL	sporadic	no	22	noradrenergic		LN, O, PR
4	M	30	PGL	1 A	SDHB	40	noradrenergic		O
5	F	68	PGL	1 A	SDHA	5	adrenergic		LN, O, PR
Mean		**47**							
StD		**17**							

*F *female, *LN *nodal, *M *male, *Mx *metastasis, *n.a. *not available, *NUP93 *nucleoporin 93, *O *osseous, *PCC *pheochromocytoma, *PGL *paraganglioma, *PR *primary, *PUL *pulmonary, *SDHA *succinate dehydrogenase subunit A, *SDHB *succinate dehydrogenase subunit B, *StD *standard deviation

### Eligibility for PRRT

In five patients, 52 individual lesions were evaluated on baseline PET (Table 2). Metastases were detected in the bone (mean SUV_max_ 41.4 ± 87.3), lungs (SUV_max_ 5.0), liver (mean SUV_max_ 24.5 ± 11.8), and lymph nodes (mean SUV_max_ 12.3 ± 9.5). Local recurrence was present in three patients (mean SUV_max_ 28.1 ± 16.5). The highest lesion-based Krenning score was 4 in three patients and 3 in two patients. Since all patients´ lesions had a Krenning score of 3 or higher, PRRT eligibility was confirmed in all cases.

**Table 2:** Baseline [^18^F]SiTATE: single lesion analysis (SUV_max_)

	Nodal (*n* = 7)	Osseous (*n* = 38)	Lung (*n* = 1)	Liver (*n* = 3)	Recurrence (*n* = 3)
Mean	12.3	41.4	5.0	24.5	28.1
StD	9.5	87.3	n.a.	11.8	16.5

*n.a. *not applicable, *StD *standard deviation

#### Response to PRRT

After completion of PRRT, all patients showed SD according to RECIST 1.1. A decrease or stable level of TTV in 2/5 (40.0%) patients was observed. CgA was stable or decreasing in all patients (Table 3). Thus, 3 patients presented with increasing TTV even though CgA was decreasing or stable and RECIST 1.1 criteria showed SD.

**Table 3:** CgA levels and imaging parameters

Baseline			1. FU			2. FU			Baseline 2		2. FU		
Pat. ID	TTV (mL)	CgA (ng/mL)	TTV (mL)	CgA (ng/mL)	RECIST 1.1	TTV (mL)	CgA (ng/mL)	RECIST 1.1	TTV (mL)	CgA (ng/mL)	TTV (mL)	CgA (ng/mL)	RECIST 1.1
1	11.0	427	31.5	309	SD	39.7	226	SD					
2	9.73	137	19.5	141	SD								
3	791	108	90.9	69.6	SD	47.2	42.4	SD					
4	29.0	109	32.1	95.0	SD								
5	564	1758	485	444	SD				181	170	250	126	SD

*BL *baseline, *CgA *chromogranin A, *FU *follow up, *n.a. *not available, *PD *progressive disease, *SD *stable disease, *TTV *total tumor volume,

* new lesions

**Fig. 2:**
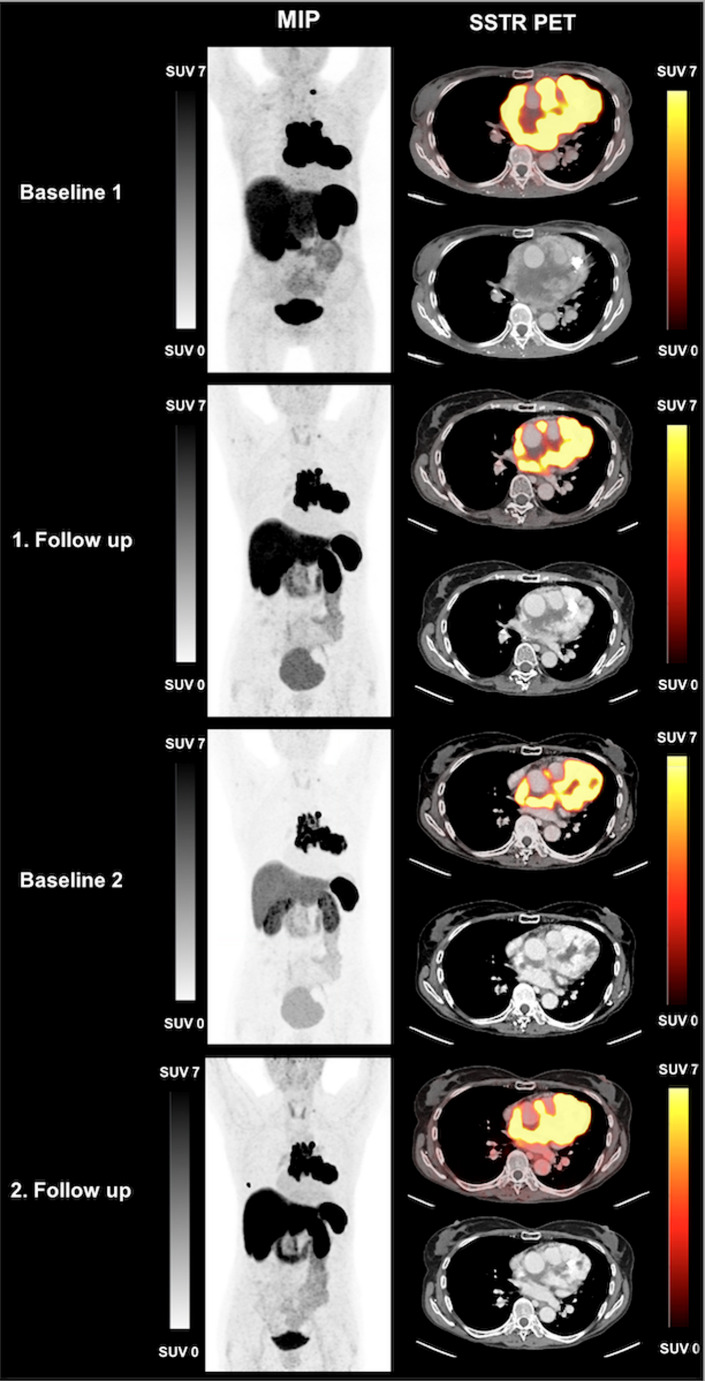
MIP and axial SSTR PET of patient #5:The 68-year-old female patient was diagnosed with thoracic paraganglioma and underwent PRRT. After two cycles of PRRT TTV decreased from 564 mL (Baseline 1) to 485 mL (1. Follow up). After one year she received the fourth cycle of PRRT (rechallenge). TTV increased from 181 mL (Baseline 2) to 250 mL (2. Follow up), RECIST 1.1 defined the response as SD. The tracer uptake above the liver in the second follow up scan presented as a contamination of the skin

The case of Patient #5 is presented in Fig. 2. She was diagnosed with a thoracic paraganglioma that encircled the pulmonary artery, infiltrated the pericardium over a considerable area and all coronary arteries. The patient Suffered from exertional dyspnea. She underwent PRRT in a rechallenge scenario and responded with a decrease of TTV and CgA level as well as SD according to RECIST 1.1 [19, 20].

## Discussion

In this retrospective analysis, we evaluated the use of the novel tracer [^18^F]SiTATE in PPGL patients, for selection of PRRT eligibility and therapy monitoring.

To date, [^18^F]SiTATE has shown excellent image quality and high lesion detectability in patients with neuroendocrine tumors, but its use for selecting candidates for PRRT in metastatic PPGL, has not been systematically studied [24, 25]. To the best of our knowledge, this is the first study evaluating [^18^F]SiTATE PET/CT for patient selection for PRRT in metastatic PPGL.

Our results indicate that [^18^F]SiTATE is effective in determining SSTR expression as shown by high image contrast in PPGL patients. A key advantage of [^18^F]SiTATE over other ^68^Ga-labelled SSTR directed tracers is its higher spatial resolution due to fluorine radiochemistry [7, 16], indicative for improved read-out capabilities of [^18^F]SiTATE. Single-lesion analysis revealed high SUV_max_ values in metastases, especially in osseous lesions.

The cohort predominantly presented with lymph node and bone metastases, while visceral metastases were only rarely observed. This distribution aligns with existing literature on metastatic patterns in PPGL [26, 27]. However, due to the low number of visceral metastases in our cohort, conclusions regarding the detection performance of [^18^F]SiTATE for visceral lesions remain limited. The high standard deviation of SUV_max_ values observed in osseous metastases likely reflects the inherent biological heterogeneity of metastatic bone involvement in PPGL, encompassing underlying genetic mutations variations and thus SSTR expression [28]. Nevertheless, the high SUV_max_ values across all detected lesions suggest [^18^F]SiTATE to be a good tracer for metastasis detection.

Interestingly, in our cohort, three patients presented with heterogenous response when comparing PET-based and CT-based assessments. Specifically, PET imaging revealed an increase of TTV, while CT-based evaluation according to RECIST 1.1 criteria indicated SD. The prognostic value of whole-body SUV_max_ in this context remains uncertain, as also reported for gastroenteropancreatic NETs [29]. The underlying reasons for this discrepancy between metabolic (PET) and morphologic (CT) response assessment remain unclear and warrant further investigation in future studies.

Hormonal biomarkers such as CgA, which is co-stored and co-secreted with catecholamines, showed inconsistent correlations with imaging-based tumor response in previous studies [5]. It has been reported, that both imaging and biochemical responses are often only seen in a Subset of patients. In contrast, our results showed a relatively high concordance between CgA levels and disease control as defined by RECIST 1.1, underscoring the potential prognostic relevance of CgA in this setting and the need for further studies to clarify its role [30, 31]. Nevertheless, CgA remains a non-specific biomarker whose levels can be influenced by various factors unrelated to tumor progression or treatment response, such as inflammation or chronic kidney disease [32–34]. Furthermore, although some studies recommend fasting measurements of CgA to reduce variability, this was not standardized in our cohort [35]. Consequently, the interpretation of CgA levels should be made cautiously and in conjunction with imaging and clinical assessment.

The potential of SSTR-positive TTV as an imaging biomarker for predicting PRRT response and its impact on progression-free survival (PFS) warrants further investigation in long-term studies. While previous research on PRRT in PPGL has yielded encouraging PFS outcomes, larger, prospective studies are essential to confirm these results and establish the prognostic significance of TTV in this specific patient population [14, 36–38]. Due to the small cohort size, PFS was not a focus of the current study but should be addressed in future research.

The small cohort is furthermore the major limitation of this study, beyond its retrospective design and heterogeneity (e.g. pretreatments and number of PRRT cycles). Subgroup analysis in regard of genetic mutations was not performed due to the limited sample size, although existing literature suggests that mutations might influence PRRT response [39]. However, the small number of patients must be viewed in the context of the rare prevalence of PPGL, even at a high-volume theranostic center, which inherently limits the availability of larger cohorts for Such studies. Although we evaluated 52 lesions in total, allowing for lesion-based observations, the limited cohort size does not permit definitive conclusions regarding diagnostic accuracy, treatment response, or prognostic value. In addition, no intra-individual comparative imaging was available using established reference tracers such as [^68^Ga]Ga-DOTATOC. As such, we cannot comment on the relative diagnostic performance of [^18^F]SiTATE compared to these modalities. The findings presented here should therefore be interpreted as preliminary and hypothesis-generating. Nonetheless, we believe this initial clinical experience provides valuable insight into the potential role of [^18^F]SiTATE PET/CT for patient selection and follow-up in the setting of PRRT for metastatic PPGL and may serve as a basis for future prospective and comparative studies. The Krenning score remains useful for assessing SSTR expression; however, its validity in SSTR PET has been questioned, since it was first established for In-111-pentetreotide SPECT in NET patients [40]. For quantitative assessment of tumor burden, we applied a fixed threshold approach for TTV segmentation to ensure consistency and reproducibility across patients and lesions. While lesion-specific or adaptive thresholds may offer more individualized volumetric measurements, they also introduce variability and reduce comparability, particularly in small exploratory cohorts [41]. The fixed threshold method has been used in a previous study on [^18^F]SiTATE PET/CT imaging and provides a practical, standardized framework for feasibility assessments [8]. Future studies with larger patient populations may benefit from more advanced segmentation strategies, including adaptive or AI-based methods. Long-term follow-up data are needed to more comprehensively evaluate the sustained efficacy and durability of PRRT beyond the short-term results presented in this study.

Despite these limitations, our findings support the use of [^18^F]SiTATE PET for patient stratification and therapy monitoring during PRRT. Nevertheless, individualized decision-making regarding continuation of therapy remains essential, particularly in light of heterogenous imaging and biochemical responses as observed in this cohort.

In summary, SSTR PET using the novel tracer [^18^F]SiTATE appears to be a feasible tool for visualizing tumor burden and supporting patient selection and treatment monitoring in metastatic PPGL undergoing PRRT. While this tracer offers several practical advantages over conventional ^68^Ga-labeled SSTR ligands, our findings are based on a small, retrospective cohort and should be interpreted accordingly. Notably, we observed discrepancies between PET-based, CT-based, and biochemical (hormonal) response assessments, the mechanisms of which remain unclear and warrant further investigation. Larger prospective studies are needed to explore the role of SSTR-positive tumor burden as a potential predictive biomarker and help elucidate the relationship between imaging and biochemical response patterns.

## Data Availability

The datasets used and/or analyzed during the current study are available from the corresponding author on reasonable request.

## Abbreviations

BL: Baseline
CgA: Chromogranin A
F: Female.
FU: Follow up.
LN: Nodal
M: Male.
n.a.: not available.
NET: Neuroendocrine tumor
NUP93: Nucleoporin 93
O: Osseous
PCC: Pheochromocytoma
PD: Progressive disease
PET: Positron emission tomography
PET: PGL Paraganglioma
PPGL: Pheochromocytoma and paraganglioma
PR: Primary
PRRT: Peptide receptor radionuclide therapy
PUL: Pulmonary
StD: Standard deviation
SD: Stable disease
SDHA: Succinate dehydrogenase subunit A
SDHB: Succinate dehydrogenase subunit B
SSTR: Somatostatin receptor
SUVSUV_max_: Maximum standardized uptake value
TTV: Total tumor volume
